# Practical Electricity in Medicine and Surgery

**Published:** 1890-07

**Authors:** 


### Book Reviews.

---

Practical Electricity in Medicine and Surgery. By G. A. Leibig, Jr., Ph. D., and George H. Rohè, M. D. Illustrated. Pages, 383. Cloth, $2.00. Philadelphia and London. F. A. Davis, Publisher, 1890.

In this volume the authors have succeeded admirably in presenting the essentials of electro-therapeutics concisely, but clearly, and with practical directness and evident candor.

The first part of the book is devoted to electricity and magnetism, the second to electro-physiology and electro-diagnosis, and the third to the application of electricity and magnetism to medical and surgical practice.

The several kinds and some of the various forms of batteries for the production of galvanism, of faradism and the galvano-cautery are fully described and freely illustrated, and the comparative merits of different machines are fairly discussed.

In the chapter on special electro-therapeutics are given the methods and the results of its use in diseases of the nervous system, of the muscles and the joints, of the eye, the ear, the nose, and of the respiratory, the circulatory and the abdominal organs.

The pages assigned to the consideration of the removal of hair by electrolysis and to the application of galvanism to the diseases of the female generative organs are particularly interesting. In reference to the former procedure, it is stated that any good constant battery will answer the demands of the operation, and that hair may be thus removed permanently, painlessly and without any resulting disfigurement. The necessary steps in the operation are carefully detailed.

The value of electricity in causing destruction of the fœtus in extra-uterine pregnancy is emphasized, and its utility in the treatment of uterine fibroids is urged with earnestness and with confidence. The reported remarkable results of Apostoli are well known, and, as the writers say, were at first received with incredulity, but "the adhesion of Sir Spencer Wells and Dr. Thomas Keith, unquestionably the foremost gynæcological surgeons living, to Apostoli's views and methods of treatment have placed the latter upon a plane where they demand the attention of all progressive practitioners."

Keith is quoted as declaring "if any one should have held firmly to hysterectomy it is myself. * * * I have, however, thrown over all surgical operations for this new treatment, and the longer I follow it the more am I satisfied. * * * What I now plead for, is that, for a time, all bloody operations for the treatment of uterine fibroids should cease, and that Dr. Apostoli's treatment, as practiced by him, should have a fair trial."

We cordially commend this work as a most trustworthy exponent of the important subject of which it treats. The type is clear and large. The illustrations are numerous, are well executed and add materially to the general excellence and to the real worth of the volume. J. B. B.

